# On the Local Structure of Water Surrounding Inorganic Anions Within Layered Double Hydroxides

**DOI:** 10.3390/molecules30081678

**Published:** 2025-04-09

**Authors:** Abderrahmane Semmeq, Kanika Anand, Antoine Carof, Adolfo Bastida, Francesca Ingrosso

**Affiliations:** 1 Laboratoire de Physique et Chimie Théoriques UMR 7019, Université de Lorraine and CNRS, F-54000 Nancy, France; 2 Departamento de Química Física, Universidad de Murcia, 30100 Murcia, Spain

**Keywords:** confinement, layered double hydroxides, molecular dynamics, water structure, solvation

## Abstract

Understanding the microscopic structure and physical–chemical properties of materials with nanoconfined domains is essential for advancing technologies in catalysis, nanomaterial design, and pharmaceutical applications. Layered double hydroxides (LDHs) are promising candidates for such innovations due to their tunable interlayer environment, which can be precisely controlled by varying the type of intercalated anion and the amount of water present. However, optimizing LDH-based technologies requires detailed insights into the local structure within the interlayer region, where complex interactions occur among anions, water molecules, and the inorganic surfaces. In this work, we present a comprehensive computational study of LDHs intercalating small inorganic anions at varying hydration levels, using atomistic molecular dynamics simulations. Our findings show good agreement with existing experimental and simulation data. We observe that monoatomic ions form either a monolayered or double-layered structures, with water molecules lying flat at low hydration and adopting more disordered configurations near the surfaces at higher hydration. In contrast, polyatomic anions exhibit distinct structural behaviors: nitrates adopt tilted orientations and form double layers at high hydration, similar to perchlorates, while carbonates consistently remain flat. Additionally, water molecules strongly interact with both anions and the surface, whereas anion–surface interactions weaken slightly as hydration increases. These results offer valuable insights into the local structural dynamics of LDHs, paving the way for more efficient design and application of these versatile materials.

## 1. Introduction

Understanding the nature of the interactions that govern how water structures itself around ions is a long-standing, fundamental question in molecular physics and chemistry. Since relatively early times, a binary picture has been proposed for this phenomenon, leading to the terms ‘structure maker’ and ‘structure breaker’, coined by Gurney [1], and the similar generalization to ions in biological systems (‘kosmotrope’ and ‘chaotrope’) [2], including a link to the Hofmeister effect. A ‘structure maker’ or ‘kosmotrope’ ion can provide an ordered environment of water molecules around them (with long-range effects), whereas ‘structure breakers’ or ‘chaotropes’ do not possess this ability [3]. However, this behavior cannot be generalized, and providing a unifying picture is still a controversial issue in the literature [4]. For instance, the organization of water around proteins in the presence of ions can be influenced by the protein structure [5,6], and ions at the water interface have their own solvation properties [7].

An additional complexity in the local environment surrounding ions in water is the effect of confinement. The structure and dynamics of electrolyte solutions in reversed micelles have been investigated and clarified at a molecular level [8,9]. In a different topology, electrolyte solutions confined between charged surfaces have received increasing attention, as they represent model systems for electrochemical devices [10].

Among media that contain a nanoconfined region, layered double hydroxides (LDHs) represent a highly versatile class of materials with promising properties for ‘green’ applications. These materials can be synthesized to exhibit low or negligible toxicity and excellent biocompatibility, making them suitable for various environmentally friendly uses [11,12,13,14,15,16]. LDHs consist of stacked inorganic sheets with a net positive charge, which are separated by interlayer spaces (interlamellar regions) containing water molecules and negatively charged ions to maintain overall charge neutrality. The anionic species that have been intercalated range from inorganic ions to organic molecules, and even large biological entities, such as proteins and DNA [17,18,19]. Given their versatility, the easiness of their preparation and their low cost, LDHs can be exploited in a wide variety of technological applications, ranging from photo- and electrocatalysis [20], depollution [21,22,23], energy storage and biomass conversion [24], and the food industry [25,26] to environmental and biomedical applications [27,28,29,30].

**Figure 1:**
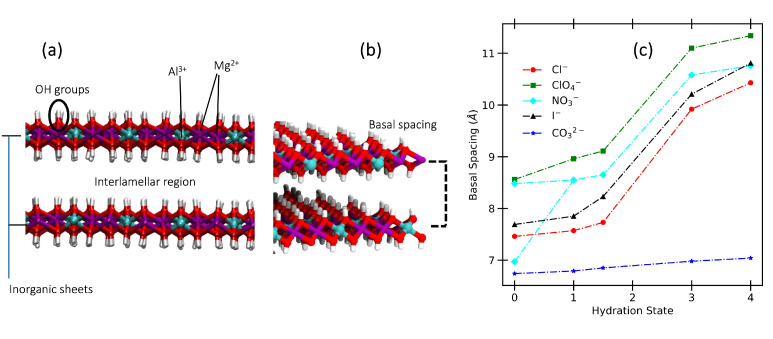
(**a**) Representation of the structure of two inorganic layers in hydrotalcite-based LDHs. Atom colors: white (H), red (O), cyan (Al), purple (Mg). (**b**) Definition of basal spacing (BS). (**c**) Average values computed along the NPT production run for the basal spacing (defined in the image on the right-hand side) in the LDHs intercalating different anions at different hydration states (i.e., number of water molecules per anion). In the case of nitrates, two values are reported at 0 H_2_O, corresponding to two different simulations, as discussed in the text.

The inorganic precursor of LDHs originates from the hydrotalcite mineral, and its composition is represented by the formula [Mg_4_Al_2_(OH)_12_]CO_3_·4H_2_O. Similarly to what is observed in the brucite mineral, octahedrally coordinated metal cations are interconnected by hydroxide (${\mathrm{OH}}^{-}$) groups within the layered inorganic sheets [11,31,32,33,34]. This general formula can be generalized to different materials by exchanging the carbonate anions in the interlamellar region with other anions, and by modifying the hydration content. As a matter of fact, changes in the amount of water confined between the inorganic sheets and surrounding the anions result in an increase in the interlamellar distance. In Figure 1a, we report an illustration of the microscopic structure of the inorganic layers, within which the interlamellar region is defined.

Among experimental studies, Iyi et al. [35] conducted the first systematic investigation of LDHs intercalating small inorganic anions (carbonates, chloride, bromide, nitrate, iodide, sulfate, perchlorate—${\mathrm{CO}}_{3}^{2-}$, Cl^−^, Br^−^, ${\mathrm{NO}}_{3}^{-}$, I^−^, ${\mathrm{SO}}_{4}^{2-}$, ${\mathrm{ClO}}_{4}^{-}$, respectively), varying the relative humidity and the Mg/Al ratio. Their findings showed that hydration increased with both charge density and anion size. Remarkably, adsorption isotherms for Li/Al and Mg/Al LDHs intercalating perchlorate, iodide, and sulfate anions exhibited two distinct steps [36,37]. For ${\mathrm{NO}}_{3}^{-}$, a reorientation likely occurs as interlamellar water content rises [38].

Advanced vibrational spectroscopy is essential for probing the local structure of water, both in bulk and within complex environments, offering a highly detailed view of the hydrogen bond network [39]. In this context, Lainé et al. [40] performed a pioneering analysis using infrared spectroscopy to study water structuring around anions at varying levels of relative humidity. Their findings revealed that at higher hydration levels, carbonate anions are bridged by water molecules, while nitrates exhibit increasingly strong water–anion interactions with hydration. The authors also proposed that both planar and tilted nitrate configurations coexist within the interlayer space. For chloride-intercalated LDHs, the authors interpreted their measurements as the result of a transition from ice-like to liquid-like water structure, beginning at 2% relative humidity. The case of perchlorate anions proved more complex: weaker hydrogen bonds were observed at lower hydration, while stronger ones appeared at higher hydration levels. This behavior could be related to the two-step shape of the hydration isotherm.

Atomistic simulations provide valuable insights into the microscopic structure of the interlayer space in LDHs under different water contents, and specific force fields (FFs) are available for clay-like materials, including hydrotalcite-based LDHs (see Refs. [41,42,43,44,45]). However, a systematic investigation of how the local water network properties change with different intercalating ions and hydration levels is still lacking.

The macroscopic behavior of LDHs depends significantly on the nature of the intercalated ions, which show varying affinities for the surface [46]. Additionally, the crystallinity, stability, and solubility of these materials differ with the type of intercalated anion [47]. Understanding how anions influence LDH phases is crucial for technological applications and for advancing knowledge of water behavior under confinement, in particular with respect to the influence of anions present in the confined environment.

This study aims to provide a consistent description of the local environment of small inorganic ions in the interlamellar region of hydrotalcite-based LDHs, with a focus on how it varies with the amount of intercalated water. We were particularly interested in unraveling specific effects related with the nature of the different anions. We conducted extensive molecular dynamics (MD) simulations on LDHs with five different small monoatomic (Cl^−^, I^−^) and polyatomic (${\mathrm{CO}}_{3}^{2-}$, ${\mathrm{NO}}_{3}^{-}$, ${\mathrm{ClO}}_{4}^{-}$) inorganic anions, for which corresponding experimental data are available in the literature. For each anion, we analyzed four hydration states in addition to the anhydrous form. We examined the structure of water through local atomic densities, molecular orientations, and radial distribution functions. We also investigated the orientations of polyatomic anions and their interactions with water and inorganic surfaces. The resulting picture is quite complex, as expected, revealing a strong dependence of the local structure on the molecular properties—such as shape and charge density—of the intercalated species.

This paper is organized as follows. Section 3 describes the computational methods used in the simulations. The results are presented and discussed in Section 2. Finally, Section 4 summarizes the findings and discusses potential implications of this work.

## 2. Results and Discussion

We start the presentation of our results by reporting the evolution of the LDH basal spacing (BS) as a function of the hydration state, for each system (Figure 1b,c). The data used to build these plots are reported as Supporting Information.

Firstly, the computed data show reasonably good agreement with experimental results reported in Ref. [35]. For the anhydrous systems, relative errors range from 0 to 10%, with the largest deviation observed for the system intercalating carbonates. It is worth noting that experimental measurements of basal spacing can vary. For instance, the value of 7.38 Å reported for the anhydrous nitrate-intercalating system in Ref. [35] differs from 8.78 Å, reported in Ref. [48].

For the hydrated states, only general trends can be discussed as a function of increasing water content. This limitation arises because experimental data are typically reported as a function of relative humidity (RH), without systematically estimating the number of water molecules per anion. An exception is represented by the system intercalating Cl^−^, for which a recent study reports that water uptake by the material can occur up to RH = 3% (corresponding to about 1.5 chlorides per water). Further increase in the RH results in capillary condensation and intercrystallite space [49]. This important remark can guide us in the comparison of the trends of the basal spacing in comparison with the work by Iyi et al. [35].

When chloride ions are intercalated, the BS values are consistent with water uptake within the layers, as in the experimental case. For 3 and 4 water molecules per anion, we observe a large expansion of the interlayer space. In the case of iodide, the experimental BS value has a large increase after RH = 60%, which corresponds to the steep rise that we observe from 1.5 H_2_O to 3 H_2_O (see Section 3 for the definition of the systems). This trend is also observed for perchlorates. Finally, in LDH intercalating carbonates, the changes in the BS values as a function of the water content are negligible, as in the experiments.

The case of nitrates warrants particular attention. The experimental change in basal spacing (BS) from the anhydrous system to higher hydration states is often interpreted as evidence of a reorientation of the anions—from lying flat and parallel to the surface to adopting a tilted, more perpendicular orientation when water is present. However, this interpretation has been challenged by Ref. [40], which shows that both parallel and tilted configurations may coexist even in dry samples. It is important to note that experiments alone cannot determine whether this coexistence occurs within the same interlayer or results from averaging across different interlayers present in the sample.

Interestingly, during the equilibration process of our simulations for the dry system, we observed three distinct scenarios. In the first scenario, the simulation box contained three interlayer regions with all nitrates in a parallel orientation, resulting in a BS of 6.97 Å. In the second scenario, all nitrates were tilted, leading to a BS of 8.09 Å. The third scenario presented a mixed configuration, with some layers containing parallel nitrates while others contained tilted ones. This observation suggests that experimental results could align with either hypothesis regarding nitrate orientation.

When water is present, nitrates reorient within the interlayer, and the experimental BS does not change significantly. The computed BS values exceeding 10 Å might therefore correspond to structural arrangements not directly observed along the water adsorption isotherm of the material.

**Figure 2:**
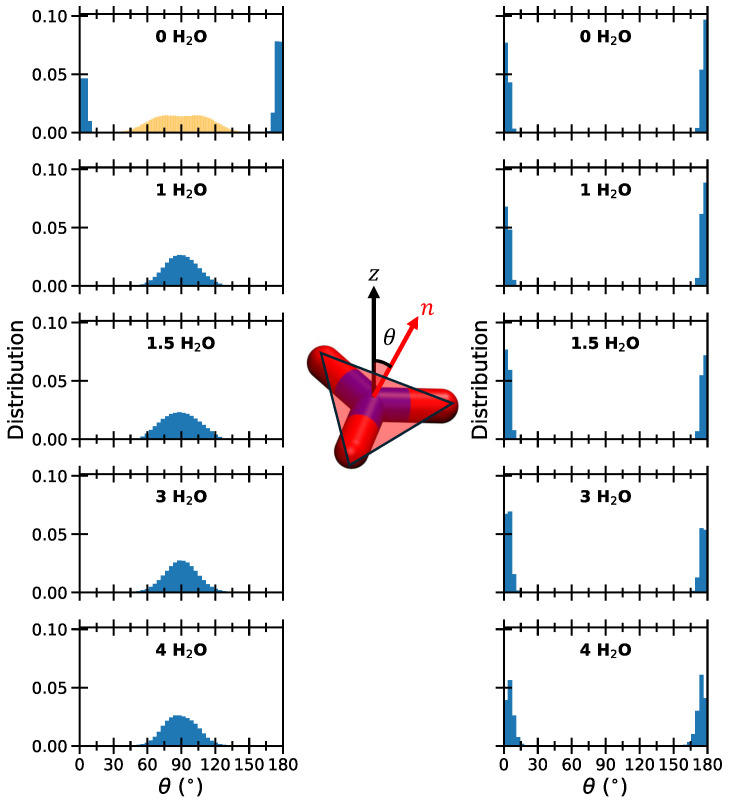
Distribution of the θ angles, defined in the inset, describing the orientation of the NO_3_^−^ (**left**) and of the CO_3_^2−^ (**right**) planes along the trajectories for the dry systems and for the different hydration states. The yellow distributions represent nitrates in a tilted configuration within the anhydrous system.

Although both carbonates and nitrates have a planar structure, their behavior as the water content increases is strikingly different, as noted in previous studies [43,44,45]. Specifically, while nitrate ions adopt a tilted orientation relative to the surface, carbonates remain flat. Consequently, in the hydration states considered in this work, the BS remains unchanged as the water content increases in the latter case. This is likely due to differences in total charge, with ${\mathrm{CO}}_{3}^{2-}$ exhibiting stronger electrostatic interactions. Another key factor is that the nitrogen-intercalated system contains one ion per unit cell, whereas the carbonate-intercalated system has one ion per two unit cells to maintain charge balance, resulting in a less crowded environment. Figure 2 shows the distribution of the angle θ, defined as the angle between the normal vector to the molecular plane and the z-axis, defined to be perpendicular to the inorganic layers.

When the water content increases, the θ distributions stay centered around 90°, as expected for tilted configurations, whereas peaky distributions are observed for carbonates, which correspond to horizontal configurations [44]. It is worth noting that, in simulation studies considering much higher hydration, carbonate anions can reorient in a perpendicular configuration as well [42]. However, this was observed when including 30 water molecules per anion, a situation that is not comparable with the hydration states considered in this work.

**Figure 3:**
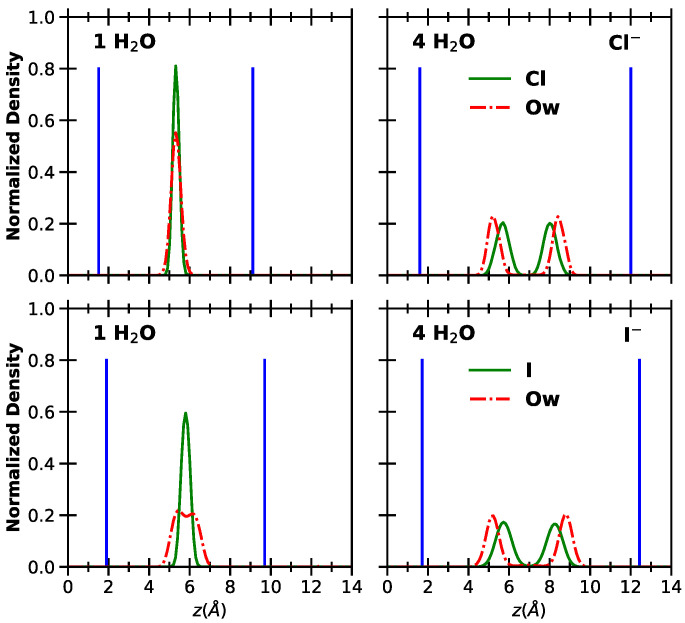
Normalized density distributions along the direction perpendicular to the layers. The *z* coordinate is computed with respect to the position of the hydroxyl groups. **Left** and **right** panels: LDH intercalating 1 water molecule and 4 water molecules per anion, respectively. **Top panel**: LDH intercalating chloride ions. **Bottom panel**: LDH intercalating iodide anions. The average positions of the Al atoms are reported in bright blue as a guide for the eye.

**Figure 4:**
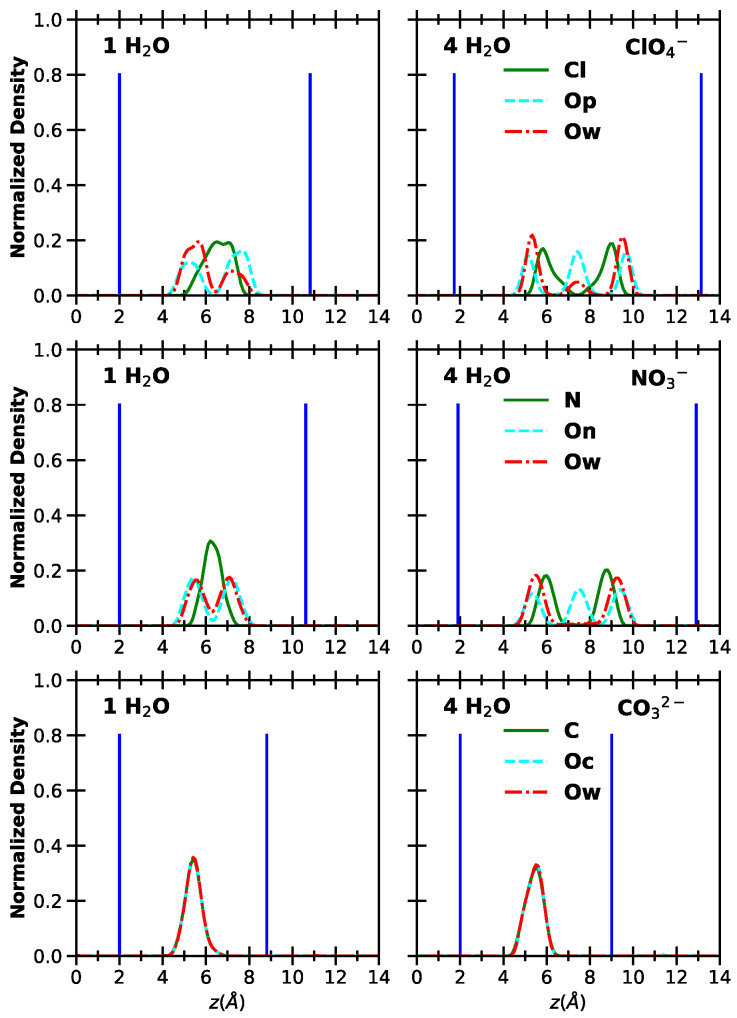
Normalized density distribution along the direction perpendicular to the layers. The *z* coordinate is computed with respect to the position of the hydroxyl groups. **Left** and **right** panels: LDH intercalating 1 water molecule and 4 water molecules per anion, respectively. From **top** to **bottom**: LDH intercalating perchlorate, nitrate and carbonate anions. Ow: O atoms of water; Op: O atoms of perchlorate; On: O atoms of nitrates; Oc: O atoms of carbonate. The average positions of the Al atoms are reported in bright blue as a guide for the eye.

To provide a more detailed description of the interlayer environment, we computed the atomic density profiles along the z-axis for relevant atoms. The results are shown in Figure 3 (for monoatomic anions) and Figure 4 (for polyatomic anions). Here, we focus on the extreme cases of 1 H_2_O and 4 H_2_O, while the full set of results is provided in the Supporting Information.

**Figure 5:**
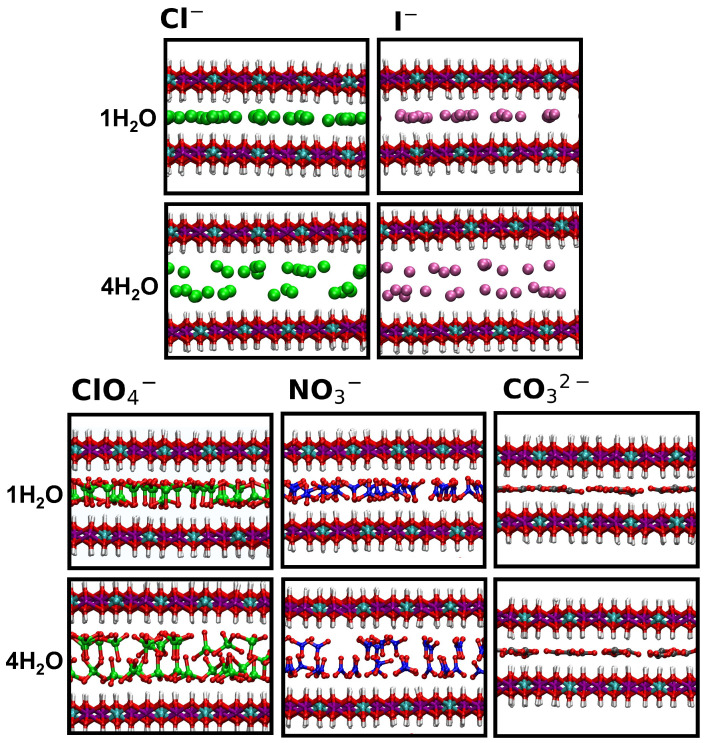
Illustration of the arrangement the anions within the LDH scaffold, depicted at low-hydration (1 H_2_O, **top**) and high-hydration (4 H_2_O, **bottom**) states. Cl^−^ anions and Cl atoms are reported in bright green, I^−^ anions in purple, C in grey, N atoms in blue, O atoms in red, H atoms in white.

The differences between LDHs intercalating chloride and iodide ions are generally small, except for a broader distribution of water molecules around I^−^ at lower hydration. In these conditions, the peaks for the oxygen atoms of water (Ow) coincide with those of the ions. In contrast, at higher hydration, two distinct peaks appear for Ow, slightly shifted from the ion positions. For Cl^−^, previous simulations suggest the formation of a flat film at low hydration and a double layer of ions surrounded by water molecules at higher hydration [41,43]. In Figure 5, we collect snapshots extracted from the simulated trajectories showing the configurations of the anions with respect to the surfaces, at lower and higher hydration. Further insights into the water structure around the ions will be discussed below.

For all systems, at low hydration, the central atoms of the polyatomic anions are located at the center of the interlayer, consistent with earlier studies [43]. At higher hydration, Cl and N atoms from perchlorates and nitrates form two symmetric peaks relative to the interlayer center, indicating the formation of a double layer. Nitrates show three peaks for the O atoms, whereas perchlorates show two. This difference arises from their molecular shape. In ${\mathrm{NO}}_{3}^{-}$, two On atoms interact with H atoms on the surface through hydrogen bonds [50], while the third On points toward the interlayer center due to the anion’s tilted planar configuration. In contrast, the tetrahedral structure of perchlorates prevents this arrangement, and the situation that is most likely to be observed correspond to anion pointing three Op atom toward the surface and the remaining one toward the center of the interlayer [42]. For carbonates, the distribution remains unchanged with hydration, showing a prominent peak for both C and Oc atoms at the center of the interlayer. These arrangements are illustrated in Figure 5.

Water molecules are located near the Op atoms of perchlorates and the On atoms of nitrates and two distributions are observed, the two peaks of which move apart when moving to higher hydration. For the system intercalating perchlorates at low hydration, an asymmetry is observed between the two peaks. Visual inspection shows that this occurs because of local deformations of the surface during the simulation, which disrupt the symmetry of the water network. Additionally, a small peak appears at the center of the interlayer for perchlorates, likely corresponding to water molecules surrounding Op atoms that point away from the surface toward the interlayer center. In contrast, for carbonates, the water peaks overlap with those of C and Oc atoms, confirming the formation of a single layer.

**Figure 6:**
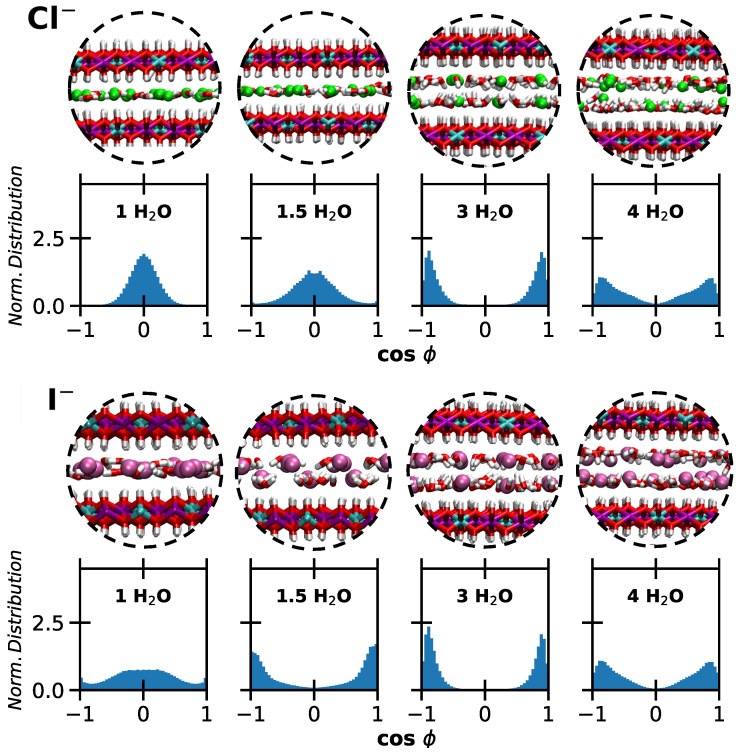
Monoatomic anions. Snapshots illustrating the interlayer region, and normalized distributions of cosϕ, describing the orientation of water molecules with respect to the *z*-axis. In the images, Cl^−^ are reported in bright green, I^−^ in purple, O atoms in red, H atoms in white. Top panels: LDH intercalating chloride ions. Bottom panels: LDH intercalating iodide anions. The intercalated water content increases from left to right.

**Figure 7:**
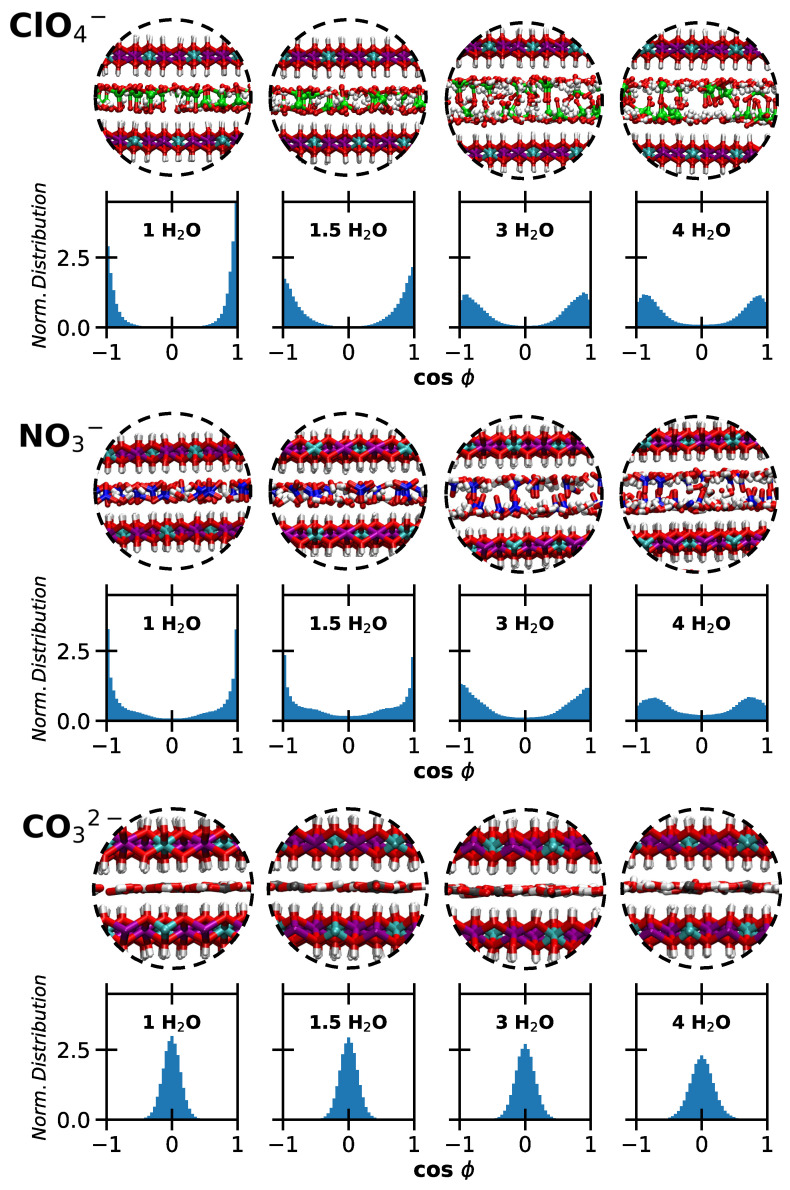
Polyatomic anions. Snapshots illustrating the interlayer region, and normalized distributions of cosϕ, describing the orientation of water molecules with respect to the *z*-axis. In the images, Cl atoms are reported in bright green, C atoms are reported in grey, N atoms in red, O atoms in red, H atoms in white. From top to bottom: LDH intercalating perchlorate, nitrate, carbonate ions. The intercalated water content increases from left to right.

**Figure 8:**
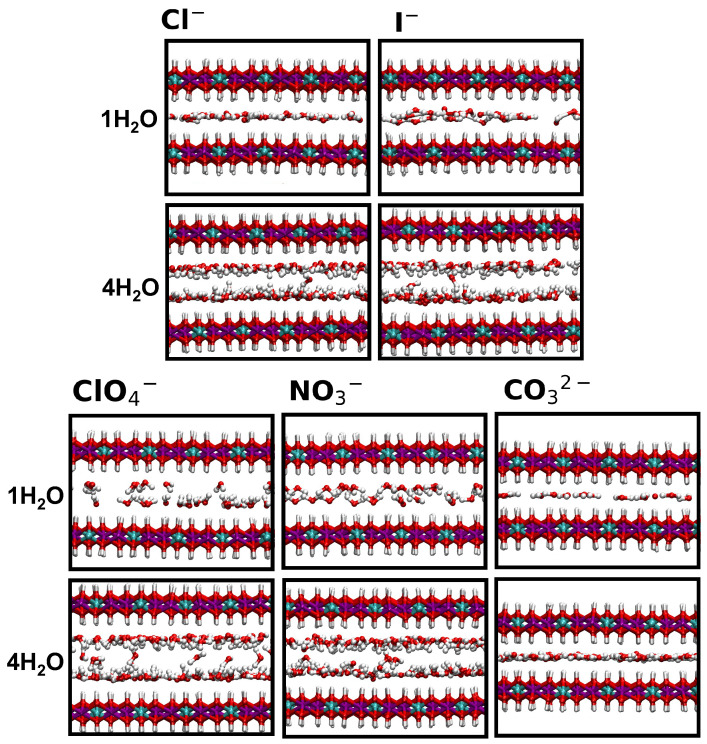
Illustration of the arrangement of water molecules within the LDH scaffold, depicted at low-hydration (1 H_2_O, **top**) and high-hydration (4 H_2_O, **bottom**) states for the LDHs intercalating different anions. O atoms are reported in red, H atoms in white.

These observations are further supported by analyzing the orientation of water molecules around the ions. Specifically, we computed cosϕ, where ϕ is the angle between the dipole moment of a water molecule and the *z*-axis. cosϕ thus represents the z component of the normalized dipole moment vector. The results are shown in Figure 6 and Figure 7, accompanied by snapshots illustrating the local structure in the interlayer. Extracted water configurations at lower and higher hydration are reported in Figure 8.

The orientation of water molecules around Cl^−^ evolves significantly with hydration. At lower hydration levels, where a single layer is observed, water molecules lie mostly flat. However, the distribution broadens as hydration increases from 1 to 1.5 H_2_O. When two layers form, there is enough space in the interlayer for water molecules to reorient, resulting in two layers adjacent to the surfaces. In this configuration, the Ow atoms point toward the surface while the hydrogen atoms (Hw) solvate the ions.

Interestingly, this reorientation occurs at all hydration states for I^−^, albeit to a limited extent at 1 H_2_O. For I^−^, the double layer forms as early as 1.5 H_2_O. Although Cl^−^ and I^−^ share the same shape and charge, the larger size of I^−^ influences the evolution of the basal spacing, as previously discussed. The radial distribution functions describing water–anion interactions (provided as Supporting Information) do not show significant differences between the two ions. Furthermore, no transition from ‘ice-like’ to ‘liquid-like’ water is observed [40], since the formation of one or two layers does not correspond to typical solid- or liquid-phase behavior.

A similar perpendicular orientation of water molecules interacting strongly with the surface is observed around perchlorates and nitrates, consistent with earlier studies [42]. Additionally, a higher degree of disorder is linked to increased water content, resulting in more varied molecular orientations. Finally, the formation of a single layer with both water molecules and carbonates lying parallel to the surfaces is confirmed at all hydration levels.

**Figure 9:**
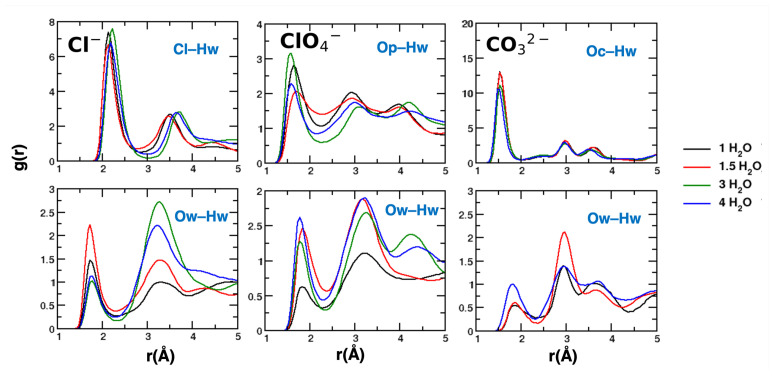
Radial distribution functions for the atom–atom interactions between anions and water (water behaving as a H-bond donor, **top panels**) and among water molecules (**bottom panels**). From left to right: LDH intercalating chloride, perchlorate and carbonate anions. Ow, Hw: O and H atoms of water. Op: O atoms of perchlorate. Oc: O atoms of carbonate.

The computed atom–atom radial distribution functions (g(r)) provide insights into key interactions, particularly those involving nearest neighbors. However, these functions inherently assume isotropic ordering, which does not apply to our confined system. For the g(r) functions computed on water molecules, intensities cannot be directly compared for different systems (e.g., the water density is different for different hydration states). Additionally, layering effects and structural constraints influence their magnitude for larger values of the distance r. Despite these limitations, valuable information can still be extracted by analyzing the position and the shape of the first peak, which reflects interactions at relatively short distances where structural constraints are most pronounced, and some of the second peaks. The most relevant findings are shown in Figure 9, while the complete set of results is provided in the Supporting Information.

**Figure 10:**
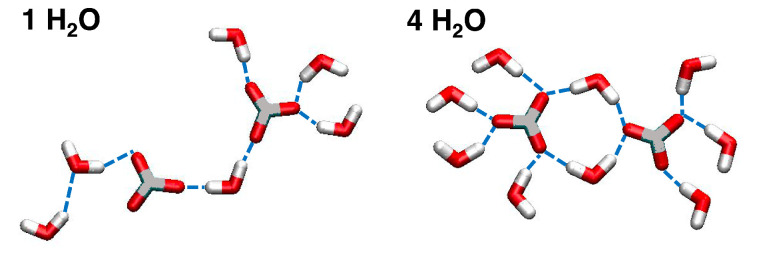
Illustration of the local hydrogen bond network for water molecules surrounding carbonate anions for two hydration states. C atoms are reported in grey, O atoms in red, H atoms in white.

Chloride ions form relatively weak hydrogen bonds with water molecules, while the Op and Oc atoms participate in stronger bonds, as indicated by the position of the first peak. For the systems forming layers (${\mathrm{Cl}}^{-}$ and ${\mathrm{CO}}_{3}^{2-}$), a significant density depletion occurs at larger distances, with additional solvation shells forming between 3 and 4 Å. Notably, the radial distribution function of Ow–Hw around carbonates shows a drastic change compared to that around chlorides, for which the radial distribution function is qualitatively more similar to that obtained for SPC/E liquid water [51]. Water molecules form strong interactions with the doubly charged ions, and it has been suggested that this leads to bridged structures, with the ions directing water molecules [40,43]. An image extracted from our simulation is reported in Figure 10 to illustrate this local structuring.

For ${\mathrm{ClO}}_{4}^{-}$, we observed a reduced intensity for the first peak of the Ow–Hw radial distribution function at 1 H_2_O, compared with the second peak. This indicates a disrupted H-bond network in the population of water molecules interacting with the surface. As the disorder increases, the water–water H-bond network is restored.

The complex environment within the interlamellar region makes it challenging to isolate and discuss the specific interactions at play. Strong Coulombic interactions arise due to the presence of ions, while a multitude of hydrogen-bonding possibilities exist—between the anions and the surface, between the anions and water molecules, among water molecules themselves, and between water molecules and the surface [41]. Moreover, as the water content increases, these interactions evolve, creating a highly interconnected network of intermolecular forces characterized by a strong collective behavior. Interestingly, a similar coexistence of donor–acceptor interactions, stabilizing the incapsulation of small molecules in a confined environment and leading to collective contributions, has been recently observed and characterized within lantern-like superphanes [52].

**Figure 11:**
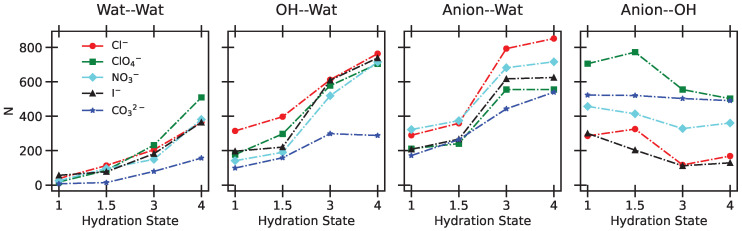
Total number of hydrogen bonds as a function of the hydration state for each studied LDH.

We decided to analyze the collective evolution of the H-bond network with the hydration state based on the total number of hydrogen bonds formed by donor–acceptor partners. Results are depicted in Figure 11 for the LDHs intercalating the different anions. Performing a similar analysis to Ref. [44], hydrogen bonds were identified using distance and angle cut-offs set at 3.5 Å for the H-bond donor–acceptor separation and 30° for the donor–H–acceptor angle, respectively.

Consistent with our previous observations, the hydrogen bond network among water molecules strengthens as the water content increases. With more water molecules present in the interlayer, they interact more with the surface, leading to an increased total number of hydrogen bonds between water and the hydroxyl groups on the surfaces. However, this interaction is relatively weak in the case of carbonates. This aligns with the formation of a single layer at all hydration states and with the presence of strong anion–water interactions, which limit the availability of water molecules to accept hydrogen bonds from the -OH groups. In contrast, for all other anions except carbonates, the anion–surface interactions decrease, although not consistently, and the water–water, hydroxyl–water, and anion–water interactions all increase sharply between 1.5 H_2_O and 3H_2_O, a behavior matching that of the basal spacing in Figure 1.

The anion–water interactions increase across all cases, with a stronger effect for carbonates. In comparing the different curves, one should keep in mind that we included 108 ${\mathrm{CO}}_{3}^{2-}$ ions in the simulation box, compared to 216 anions in the other cases.

## 3. Materialsand Methods

Classical MD simulations were performed using LAMMPS (24 Mar 2022) [53] and visualized by means of VMD [54]. For the inorganic surfaces, the modified DREIDING force field was used [55], while the parameters for the anions were adopted from Smith et al. for Cl^−^ [56], Baaden et al. for ${\mathrm{ClO}}_{4}^{-}$ [57], Cadena et al. for ${\mathrm{NO}}_{3}^{-}$ [58], Schmid et al. for ${\mathrm{CO}}_{3}^{2-}$ [59] and Blazkez et al. for I^−^ [60]. The intercalated water molecules were described using the SPC/E model [61].

For the LDHs intercalating each anion, five hydration states were considered, named 0 H_2_O, 1 H_2_O, 1.5 H_2_O, 3 H_2_O, and 4 H_2_O, with the integer indicating the number of water molecules per anion. We calibrated the choice of our hydration states to explore the range of measured basal spaces for the systems of choice, published in Ref. [36].

Simulations were run in parallelepipedal boxes and periodic boundary conditions were taken into account with the particle–particle particle–mesh solver for treating long-range electrostatics [62], whereas Lennard–Jones intermolecular interactions were cut off at half of the simulation box in the three directions. Simulation boxes comprised three inorganic layers and three interlamellar regions, to increase statistics [41]. In each of the three inorganic sheets, we included 72 unit cells, leading to a total of 216 anions (for LDHs intercalating monovalent anions) and 108 anions (in the case of carbonates). Average simulation sizes are reported as Supporting Information.

We employed a multi-step equilibration protocol to accurately model the complex chemical environment. Due to the strong electrostatic interactions present in the system, intercalated anions can become trapped in local energy minima, heavily influenced by the initial configuration. This can result in uneven concentration distributions within the LDH scaffold, and separation into anion- and water-rich zones as a result of poor equilibration.

To overcome these challenges, we adopted an equilibration protocol starting from random initial configurations generated using Packmol [63] at low density, ensuring expanded interlamellar spacing. Water molecules and anions were randomly placed within the system and initially optimized using the conjugate gradient algorithm, while the substrate was kept fixed.

The system was gradually heated to ambient temperature (T = 298 K) at a rate of 0.3 K/ps over a 1 ns simulation using the Nose–Hoover thermostat [64] within the constant volume and temperature ensemble (NVT). This was followed by an additional 1 ns of temperature equilibration. A 1 fs integration time step was used for all simulations with the velocity–Verlet integrator, and the SHAKE algorithm was applied to constrain all O–H stretching in water and hydroxide layers [65].

Next, three NVT annealing cycles were performed, each involving heating the system to 800 K and gradually cooling it back to 298 K over 10 ns. This approach minimized the risk of the system becoming trapped in a local energy minimum influenced by the initial configuration.

The system was then compressed within the constant-pressure and -temperature ensemble (NPT) over 500 ps at 500 K using the Nose–Hoover barostat [64]. A final 1 ns NPT equilibration step ensured proper density, temperature, and pressure conditions, allowing for the relaxation of residual stresses.

Lastly, 100 ns production runs were conducted within the NPT ensemble. Data analysis was performed on the production trajectories using a combination of post-processing tools available within the LAMMPS package and custom-developed in-house tools.

## 4. Conclusions

In this work, we conducted 100 ns MD simulations of LDHs intercalating five small inorganic anions at different hydration states, to shed light on the structural properties of the disordered, nanoconfined region between the inorganic layers of clay-like materials. Our findings do not reveal a unified behavior. Each system exhibits unique structural characteristics concerning the position and orientation of the intercalated anions and the surrounding water molecules. The conclusions that we were able to draw are in agreement with previous experimental and theoretical reports, when available [35,40,41,44,45,48,49].

The system intercalating carbonates shows a particularly distinct behavior. In both the dry state and at all hydration levels, carbonates orient parallel to the surface, forming strong interactions with it. These doubly charged anions also interact strongly with water molecules, disrupting the water–water hydrogen bond network.

Nitrates adopted a parallel orientation only in the anhydrous state, but stable simulations also showed tilted configurations and mixed scenarios, where some interlayers within the same simulation box contained parallel nitrates while others were tilted. Upon hydration, nitrates consistently shift to a tilted (near-perpendicular) orientation, and a double layer is formed at higher hydration. At low hydration, water molecules align next to the surface with their symmetry axis perpendicular to it, allowing the oxygen atom to accept H-bonds from the surface. This pattern is also observed with perchlorates, where the structured arrangement significantly weakens the nearest-neighbor water–water interactions. As hydration increases, a second population of more disordered water molecules appears within the interlamellar space, strengthening water–water interactions.

For the monoatomic ions Cl^−^ and I^−^, a single layer of water molecules forms parallel to the surface at low hydration. This layer is more disordered around the larger iodide ion. As the water content increases, the single layer transitions into a double layer, accompanied by the emergence of water molecules adjacent to the surface that accept hydrogen bonds from it.

This systematic investigation of the interlayer structure of LDHs provides valuable insights into the structure–property relationship in these systems. We have elucidated how confined water behaves differently depending on the intercalated anion. This behavior occurs within a layered topology, which contrasts with the more widely studied spherical confinement observed in systems like reverse micelles [8,9]. These results contribute to advancing our fundamental understanding of nanoconfined water and ion behavior in LDHs.

Extending this approach, future work will focus on exploring dynamic properties, an area already yielding intriguing findings. For example, simulations of LDHs intercalating chloride ions revealed that water diffuses using a mechanism that is similar to atomic movement in a solid lattice [66]. In addition, recent experimental studies have shown that the conductivity of -OH groups in LDHs intercalating these small inorganic ions aligns with the lyotropic series [45]. Gaining a deeper microscopic understanding of this phenomenon could enhance the design and optimization of synthetic strategies for potential applications in electrochemical devices.

## Data Availability

The original contributions presented in this study are included in the article/Supplementary Material. Further inquiries can be directed to the corresponding authors.

## Supplementary Materials

The following supporting information can be downloaded at https://www.mdpi.com/article/10.3390/molecules30081678/s1. The details about the simulation boxes, the basal spacings, the cosθ distributions of NO_3_^−^ and CO_3_^2−^, and the complete results obtained for the radial distribution functions and for the atomic distributions along the z-axis are provided as Supporting Information.
