# Supplementary material for: On the Local Structure of Water Surrounding Inorganic Anions Within Layered Double Hydroxides
Source: Molecules. 2025 Apr 9;30(8):1678. doi: 10.3390/molecules30081678 (PMC12029850; doi:10.3390/molecules30081678)
Supplement: Supplementary file 1 [file molecules-30-01678-s001.zip › molecules-3526116-supplementary.pdf]

Supporting Information for: On the Local Structure of Water Surrounding Inorganic Anions Within Layered Double Hydroxides

Abderrahmane Semmeq[1],* Kanika Anand,[1] Antoine Carof,[1] Adolfo Bastida[2], and Francesca Ingrosso[1]*

[1] *Laboratoire de Physique et Chimie Th´eoriques UMR 7019, Université de Lorraine and CNRS, F-54000 Nancy, France*

[2] *Departamento de Química Física, Universidad de Murcia, 30100 Murcia, Spain*

Correspondence: a.semmeq@gmail.com (A.S.); francesca.ingrosso@univ-lorraine.fr (F.I.)

In the following pages, we report the details about the simulation box sizes, the distributions describing the orientation of nitrate and carbonate anions as a function of the $cos\ \theta$ (with θ defined in the manuscript), and the complete collection of results for basal spacings, for z-density profiles and radial distribution functions.

Table S1: Average values obtained for the simulation box size along the NPT production runs, for LDHs intercalating different anions and at different hydration states. The standard deviation ranges from 0.03 Å to 0.05 Å

Water molecules / anion:	0	1	1.5	3	4
	x = 32.44 Å	x = 32.53 Å	x = 32.59 Å	x = 32.50 Å	x = 32.53 Å
Cl^-	y = 56.19 Å	y = 56.35 Å	y = 56.34 Å	y = 56.29 Å	y = 56.41 Å
	z = 22.33 Å	z = 22.70 Å	z = 23.13 Å	z = 29.78 Å	z = 31.28 Å
	x = 32.49 Å	x = 32.50 Å	x = 32.52 Å	x = 32.51 Å	x = 32.53 Å
ClO_4^-	y = 56.25 Å	y = 56.29 Å	y = 56.30 Å	y = 56.34 Å	y = 56.43 Å
	z = 25.70 Å	z = 26.95 Å	z = 27.23 Å	z = 33.27 Å	z = 34.07 Å
	x = 32.51 Å	x = 32.56 Å	x = 32.47 Å	x = 32.46 Å	x = 32.50 Å
NO_3^-	y = 56.31 Å	y = 56.21 Å	y = 56.22 Å	y = 56.19 Å	y = 56.30 Å
	z = 20.90 Å	z = 25.66 Å	z = 25.89 Å	z = 31.75 Å	z = 32.23 Å
	x = 32.43 Å	x = 32.54 Å	x = 32.55 Å	x = 32.53 Å	x = 32.50 Å
I^-	y = 56.21 Å	y = 56.44 Å	y = 56.14 Å	y = 56.28 Å	y = 56.39 Å
	z = 23.04 Å	z = 23.52 Å	z = 26.22 Å	z = 30.66 Å	z = 32.29 Å
	x = 32.38 Å	x = 32.34 Å	x = 32.39 Å	x = 32.36 Å	x = 32.49 Å
CO_3^{2-}	y = 56.07 Å	y = 56.04 Å	y = 56.08 Å	y = 56.11 Å	y = 56.21 Å
	z = 20.18 Å	z = 20.47 Å	z = 20.54 Å	z = 20.93 Å	z = 21.16 Å

Table S2: Basal spacing measured as an average along the NPT production run for the different systems studied. The standard deviation ranges from 0.1 Å to 0.8 Å

Water molecules / anion	0	1	1.5	3	4
Cl^-	7.46 Å	7.57 Å	7.73 Å	9.92 Å	10.43 Å
ClO_4^-	8.56 Å	8.96 Å	9.11 Å	11.10 Å	11.34 Å
NO_3^-	6.97,8.09 Å	8.55 Å	8.65 Å	10.58 Å	10.76 Å
I^-	7.69 Å	7.85 Å	8.23 Å	10.21 Å	10.81Å
CO_3^{2-}	6.74 Å	6.79 Å	6.85 Å	6.98 Å	7.04 Å

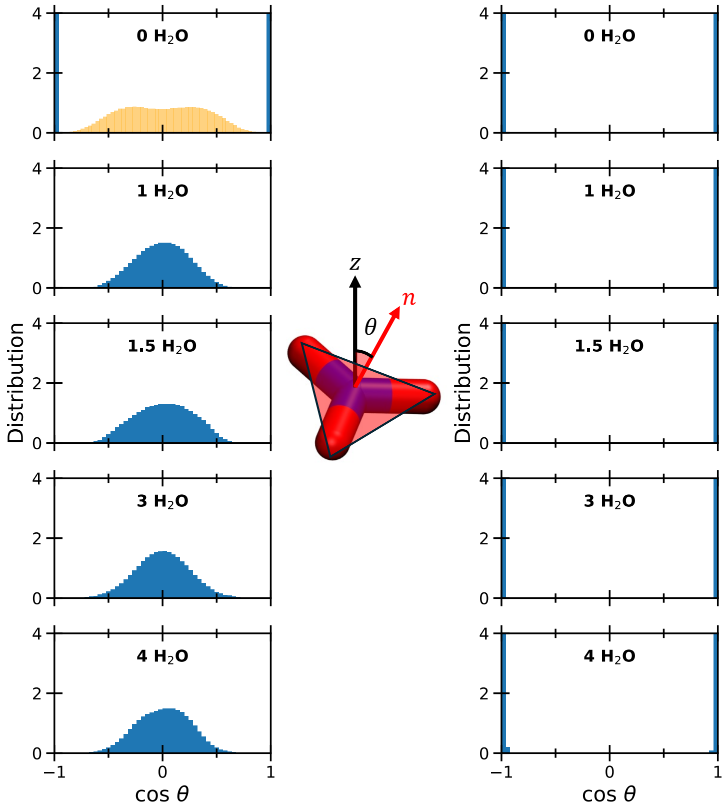

Figure S1: Distribution of the *cos* θ, where θ defined in the inset, describing the orientation of NO_3^- (left) and CO_3^{2-} (right) along the trajectories for the dry systems and for the different hydration states. The yellow distributions represent nitrates in a tilted configuration within the anhydrous system.

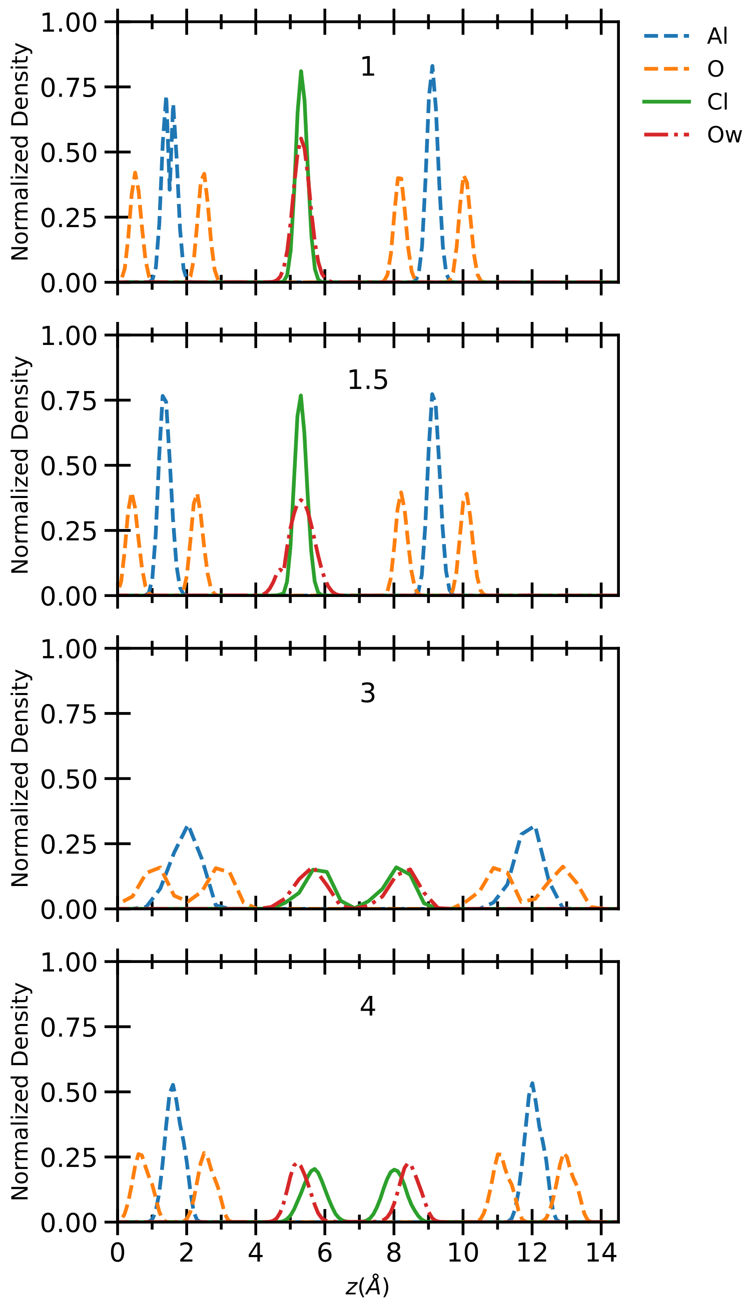

Figure S2: Density profile in z-direction for relevant atoms in the LDH intercalating chloride ions at the different hydration states (1—4: number of water molecules per anion).

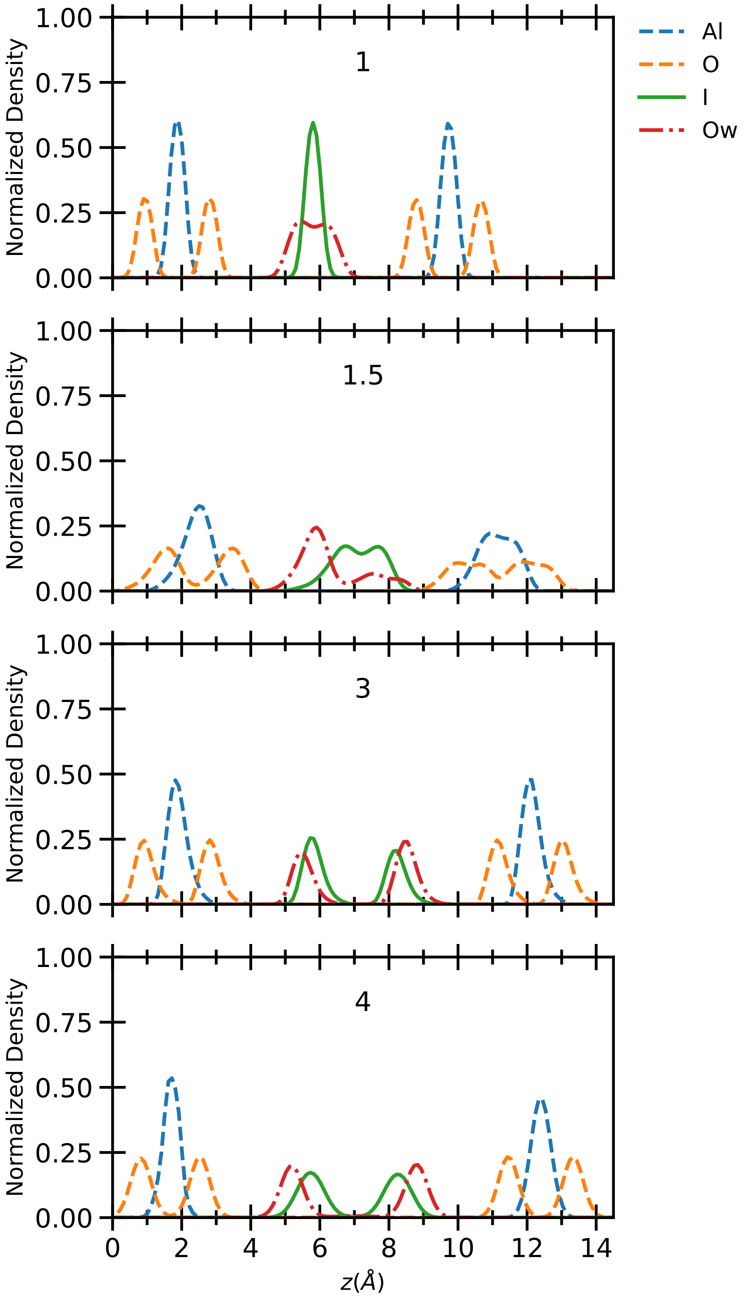

Figure S3: Density profile in z-direction for relevant atoms in the LDH intercalating iodide ions at the different hydration states (1—4: number of water molecules per anion).

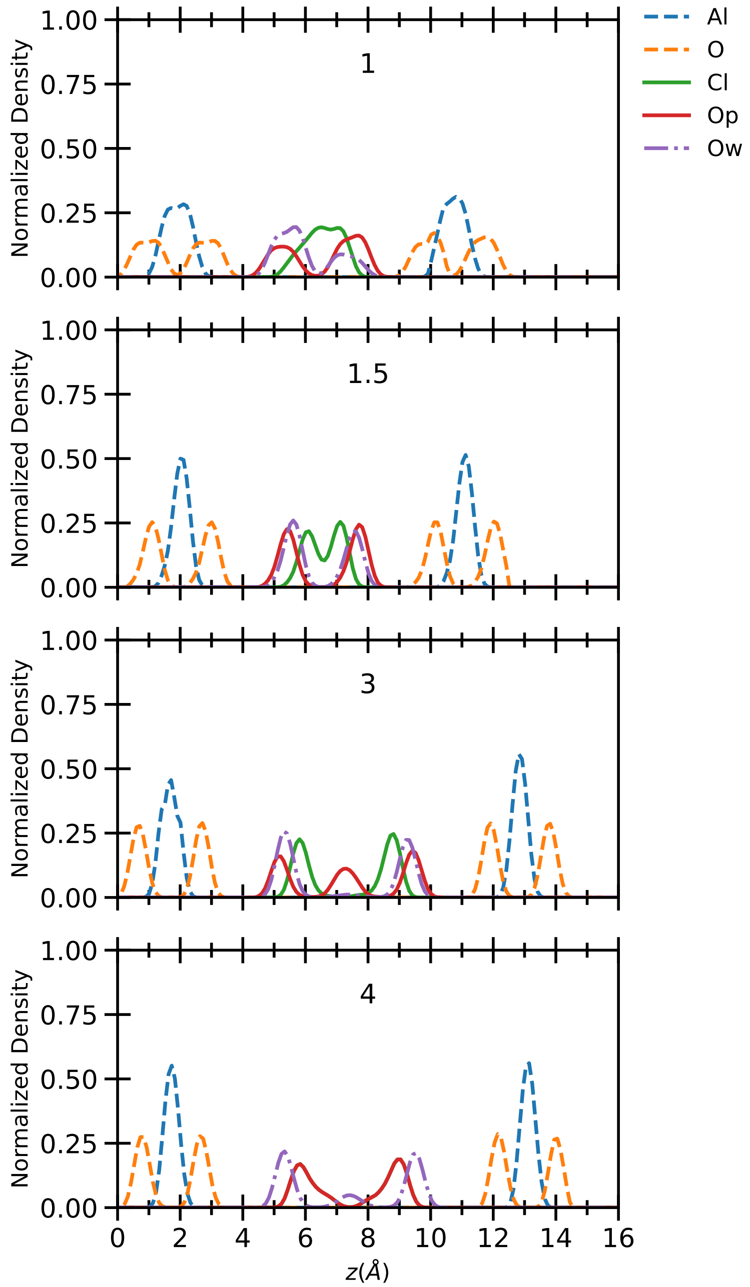

Figure S4: Density profile in z-direction for relevant atoms in the LDH intercalating perchlorate ions at the different hydration states (1—4: number of water molecules per anion).

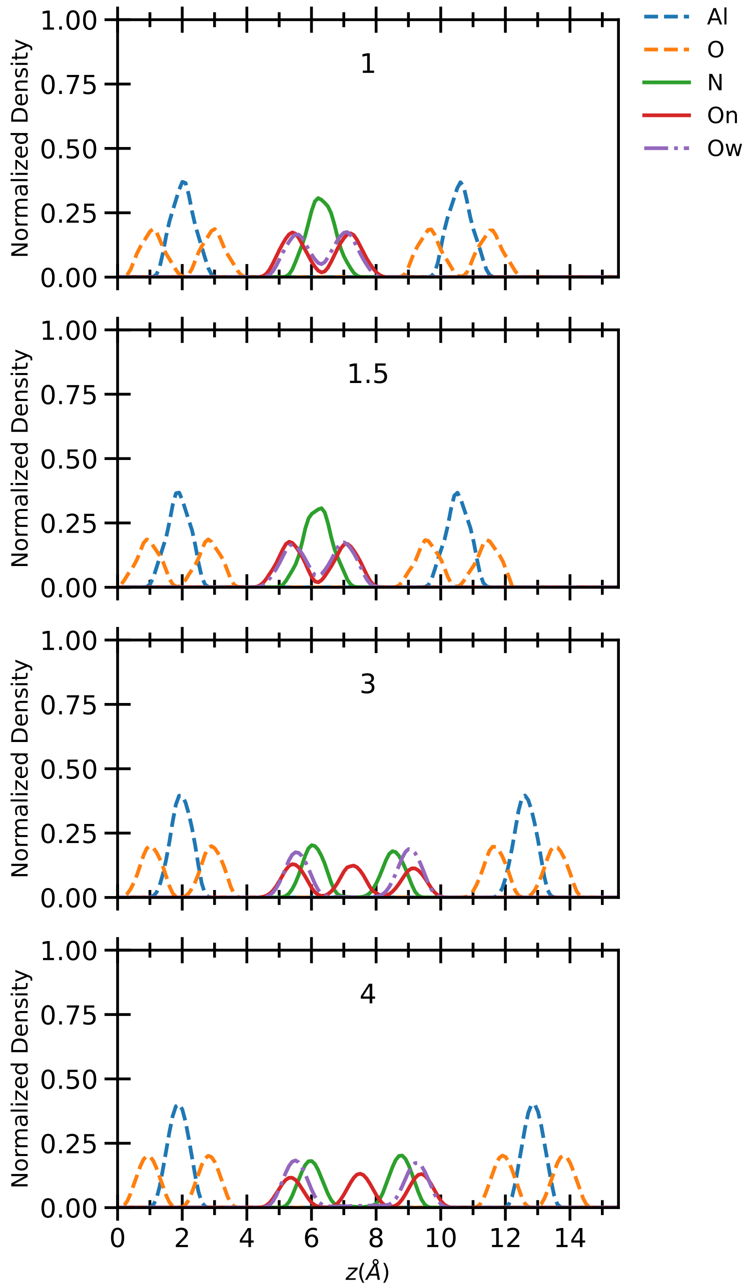

Figure S5: Density profile in z-direction for relevant atoms in the LDH intercalating nitrate ions at the different hydration states (1—4: number of water molecules per anion).

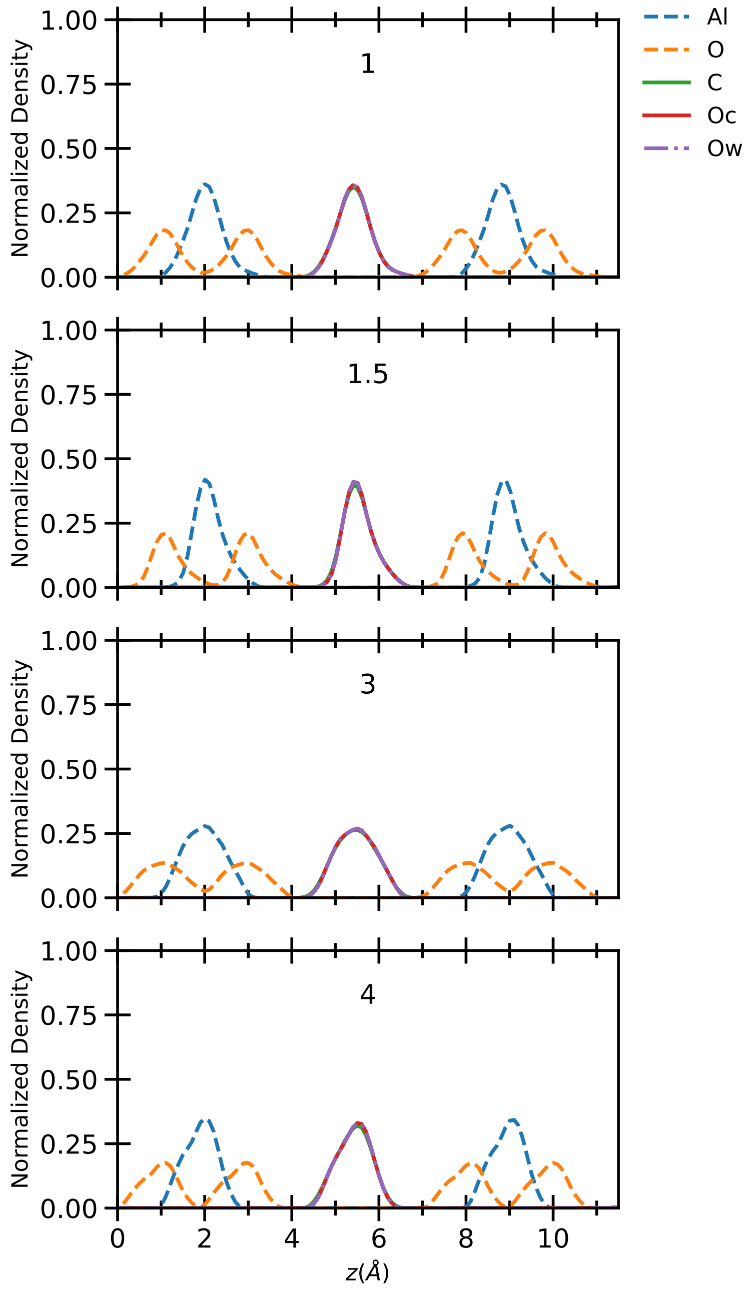

Figure S6: Density profile in z-direction for relevant atoms in the LDH intercalating carbonate ions at the different hydration states (1—4: number of water molecules per anion).

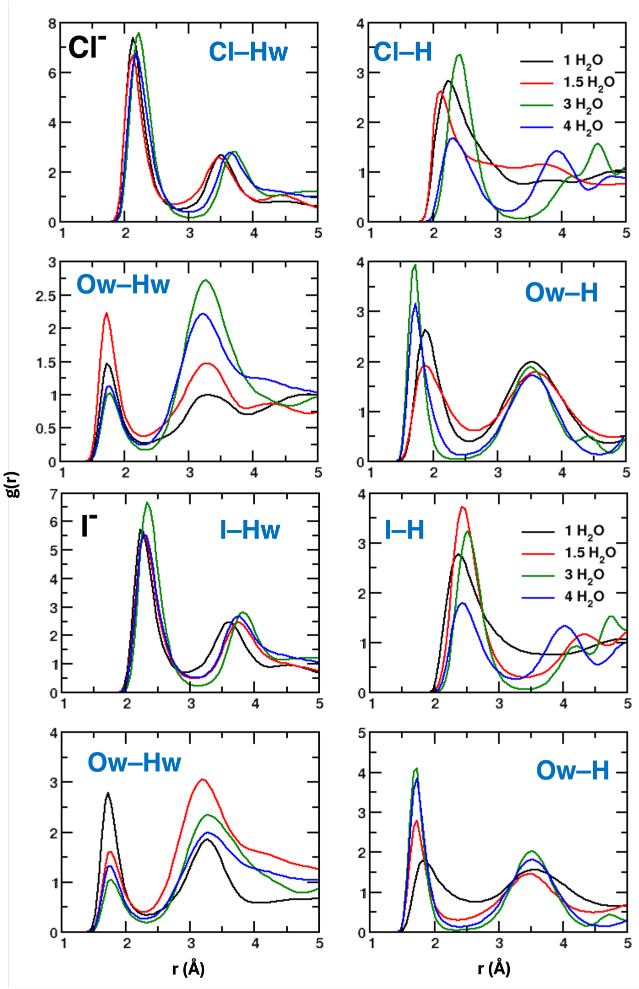

Figure S7: Monoatomic anions: radial distribution functions for relevant atoms in the interlamellar region of LDH at different hydration states. The investigated atom-atom interactions are indicated in each plot (blue text).

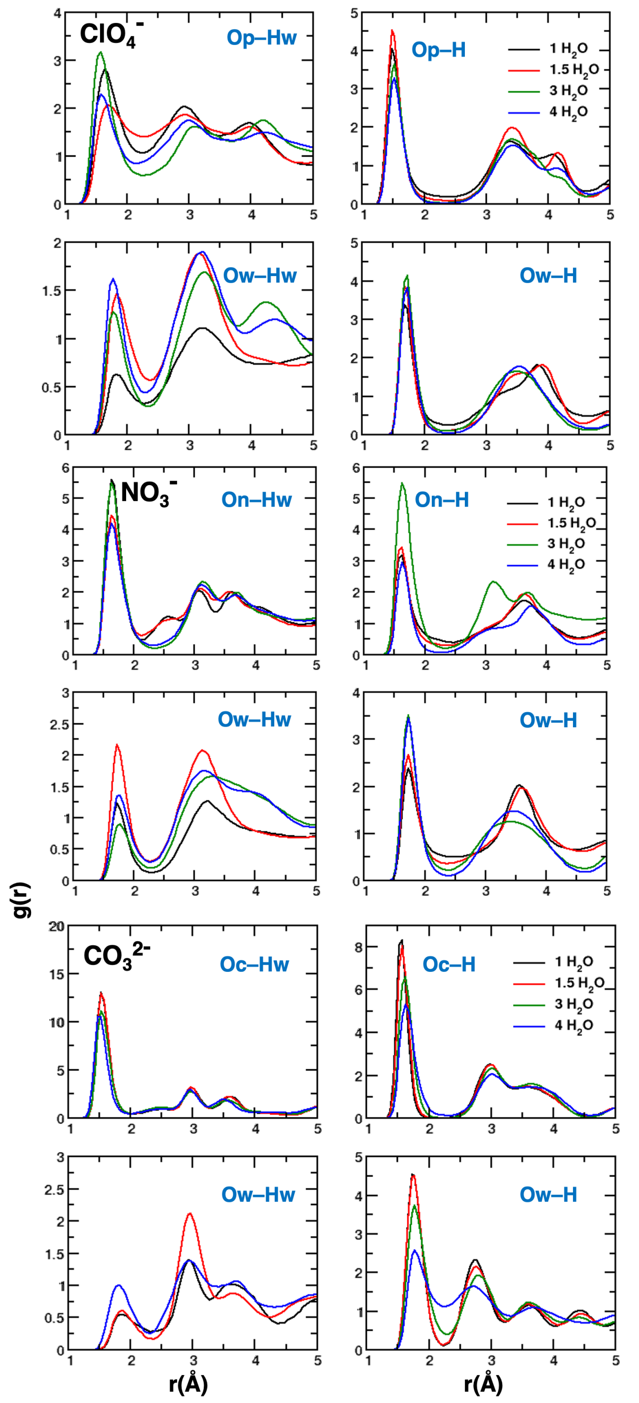

Figure S8: Polyatomic anions: radial distribution functions for relevant atoms in the interlamellar region of LDH at different hydration states. The investigated atom-atom interactions are indicated in each plot (blue text).
